# Player Tracking Data Analytics as a Tool for Physical Performance Management in Football: A Case Study from Chelsea Football Club Academy

**DOI:** 10.3390/sports6040130

**Published:** 2018-10-26

**Authors:** Varuna De Silva, Mike Caine, James Skinner, Safak Dogan, Ahmet Kondoz, Tilson Peter, Elliott Axtell, Matt Birnie, Ben Smith

**Affiliations:** 1 Loughborough University London, Loughborough University, London E15 2GZ, UK; 2 Chelsea Football Club Academy, Cobham KT11 3PT, UK; Tilson.Peter@ChelseaFC.com (T.P.); Elliott.Axtell@ChelseaFC.com (E.A.); Matt.Birnie@ChelseaFC.com (M.B.); Ben.Smith@ChelseaFC.com (B.S.)

**Keywords:** sports analytics, player tracking, football (soccer)

## Abstract

Background: Global positioning system (GPS) based player movement tracking data are widely used by professional football (soccer) clubs and academies to provide insight into activity demands during training and competitive matches. However, the use of movement tracking data to inform the design of training programmes is still an open research question. Objectives: The objective of this study is to analyse player tracking data to understand activity level differences between training and match sessions, with respect to different playing positions. Methods: This study analyses the per-session summary of historical movement data collected through GPS tracking to profile high-speed running activity as well as distance covered during training sessions as a whole and competitive matches. We utilise 20,913 data points collected from 53 football players aged between 18 and 23 at an elite football academy across four full seasons (2014–2018). Through ANOVA analysis and probability distribution analysis, we compare the activity demands, measured by the number of high-speed runs, the amount of high-speed distance, and distance covered by players in key playing positions, such as Central Midfielders, Full Backs, and Centre Forwards. Results and Implications: While there are significant positional differences in physical activity demands during competitive matches, the physical activity levels during training sessions do not show positional variations. In matches, the Centre Forwards face the highest demand for High Speed Runs (HSRs), compared to Central Midfielders and Full Backs. However, on average the Central Midfielders tend to cover more distance than Centre Forwards and Full Backs. An increase in high-speed work demand in matches and training over the past four seasons, also shown by a gradual change in the extreme values of high-speed running activity, was also found. This large-scale, longitudinal study makes an important contribution to the literature, providing novel insights from an elite performance environment about the relationship between player activity levels during training and match play, and how these vary by playing position.

## 1. Introduction

Performance management of elite soccer players is a complex process that involves optimizing their physical performance, skill-based training, tactical training, minimizing risk of injuries, and providing psychological support [1]. Achieving optimal physical performance and minimizing risk of injury can be in conflict, since improving physical performance often necessitates intensive exercise regimes which may increase the likelihood of player fatigue and thus risk of injury. Managing training loads is crucial in enabling players to physically perform at an optimal level across the duration of a playing season.

Analysis of player tracking data has gained popularity as a performance analysis tool in sports [2], especially in soccer [3,4,5,6]. There are two main types of position tracking technology: the multiple camera method and global positioning system (GPS) method [7]. Both methods have been utilised for performance analysis in many professional sports such as rugby [8], Australian rules football, hockey [9,10], and soccer [11,12]. The Global Positioning System (GPS) is a navigational system that uses information sent by multiple satellites to calculate the geographical position of a receiver (i.e., longitude, latitude, and altitude) [13]. The modern GPS receivers have integrated triaxial accelerometers, which measure the acceleration in three planes to produce a composite vector magnitude known as G-Force [14]. The integrated accelerometers can be used to quantify the forces acting on the player, known as body load, and can also be used to measure impact of players with other objects and surfaces [15].

Player tracking in soccer enables monitoring of training load, thus assisting practitioners to determine whether an athlete or a team is adapting to the training programme applied and to minimize the risk of fatigue and injury [16]. Di Salvo et al. analysed the mean distance covered at different intensities (speeds) through their study of 300 elite soccer players during match play and observed significant differences based on playing positions [3]. Bradley et al. discussed the positional variations of high-intensity running patterns of elite soccer players in the English Premier League [4]. Barnes et al. studied seasonal variations of physical demand on premier league football players, concluding that the number of sprints and sprint distance increased during seven seasons between 2006 and 2013 [5]. Bush et al. discussed the position-specific evolution of physical performance parameters in over seven seasons of Premier League football and argued that evolving tactics paved way for change in physical demands at different positions in [6]. The high-intensity running demands in three competitions played in by English professional soccer teams (League 1, Premier League, and Champions League), were deemed to be different across the competitions, with respect to total distances covered at different intensities [17], and players at lower competitive standards requiring greater high-speed running distances [18]. Hewitt, Norton, and Lyons analysed the movement pattern variations with respect to opposition ranking for elite women soccer players [19]. In summary, there is a substantial body of literature pertaining to physical activity demands analysed through tracking data of elite soccer players during competitive matches, with a specific focus on high-speed running activities and positional variations. However, there is minimal analysis relating to training demands in soccer, and any relationship between competitive matches and training regimes. Furthermore, an optimal model to manipulate training loads in order to achieve peak match performance has yet to be fully established. Many authors propose future work to analyse performance tracking data during competitive games to develop specific training programmes [4,6,19]. If patterns between training and match demands are known, it may assist with developing scientifically rigorous training programmes which integrates all aspects of training such as technical, tactical, and physical.

By utilizing retrospective analysis of tracking data, this paper investigates the relationships between physical demands during training sessions and competitive matches at an elite soccer academy, which takes a step towards developing an evidence-based approach in this area. GPS based player tracking data from four seasons has been analysed in this investigation. The aim of this study is to compare the physical activity demands in soccer between competitive matches and the training sessions across four seasons, with particular focus on positions of play.

## 2. Methods

### 2.1. Data Source

Data was collected and analysed from 150 different players, aged between 18 and 23 across four seasons (2014–2018). The summary of the data set is presented in Table 1. For the analysis, three distinct positions of play were selected: Centre Forwards (CFs), Central Midfielders (CMs), and Full Backs (FBs). The utilisation of the data for research purposes was formally approved by the Ethics Committee of the academic researchers’ institution. In approving the study the Committee were satisfied that the processes put in place by the Football Academy (which did not include obtaining written consent) ensured that the Academy players were informed about the collection of the GPS data and the uses to which these data would be put. The data are anonymised prior to analysis and aggregated such that it is not possible to link any of the data presented to any given player.

### 2.2. Acquisition of Data

In our research, 20,913 entries of data in total were analysed. Each entry corresponds to a summary of performance of one player in one day. While in a given season, a certain player predominantly trains in one squad (e.g., U18); it is common for the player to have training sessions in different squads (e.g., U16/U21). Due to this reason, for some players, the number of data entries per year does not equate to number of entries in a full season of play.

To acquire the data needed, a wearable GPS tracking unit (Model: SPI-HPU) from GPSports Australia was used, which tracked the movements of each player along with their heart rate during all training sessions and match days. The performance of the GPS tracking unit used in this study has been independently validated in a number of studies [20,21,22]. The movement data were then processed to produce metrics of physical performance for each collection period. There is a ‘data entry’ for each player for each session of play during the playing seasons. For the majority of the days, there is one session of activity per day, with few entries of two sessions of activity per day. A single data entry consists of multiple fields such as the date, season, training availability, type of day (match or training), duration of play, anonymised player code, position of play, squad, competition, and several physical performance metrics. The physical performance metrics collected are described in the next subsection.

### 2.3. Description of the Physical Performance Metrics

The number of high-speed runs and the high-speed distance (i.e., the distance covered during high-speed runs) are the most commonly utilised physical performance measures reported in recent literature [4,5,23]. Furthermore, some studies profiled running behaviour at different intensities. For example in a previous paper [24], any movement between 21.1 kmph and 24 kmph is considered a high-intensity run, and a movement above 24 kmph is considered a sprint. The current study is focused on the differences between training and match sessions. Therefore, only the high-speed running events are profiled and not running at different intensities. For the purpose of this study, any movement beyond 21 kmph is considered to be a High Speed Run (HSR).

Two different types of performance metrics have been utilised for the analysis in this paper. They are the number of HSRs above 21 kmph, and the distance covered in high-speed running. Each entry in the data set corresponds to different durations of play, because they correspond to both matches and training sessions. Therefore, to utilise as much of the data as possible in this analysis, the number of high-speed runs and the distance covered during high-speed runs have been normalised by the duration of activity for each day. Therefore, the two metrics used in this work is denoted as HSR21pMIN (for the number of HSRs above 21 kmph per minute of play) and DIST21pMIN (for high-speed distances per minute of play), and can be calculated as follows:

(1) $$\mathrm{HSR}21\mathrm{pMIN}=\frac{\mathrm{Number}\;\mathrm{of}\;\mathrm{HSRs}\;\mathrm{on}\;a\;\mathrm{given}\;\mathrm{day}}{\mathrm{Duration}\;\mathrm{of}\;\mathrm{activity}\;\mathrm{during}\;\mathrm{the}\;\mathrm{day}\;\left(\mathrm{minutes}\right)}$$

(2) $$\mathrm{DIST}21\mathrm{pMIN}=\frac{\mathrm{Distance}\;\mathrm{covered}\;\mathrm{at}\;\mathrm{more}\;\mathrm{than}\;21\;\mathrm{kmph}\;\mathrm{on}\;a\;\mathrm{given}\;\mathrm{day}}{\mathrm{Duration}\;\mathrm{of}\;\mathrm{activity}\;\mathrm{during}\;\mathrm{the}\;\mathrm{day}\;\left(\mathrm{minutes}\right)}$$

By normalising the number of events of high-speed running and the distance covered in HSRs by the duration of activity (training or matches) during a given day, we obtain a measure of the frequency of HSRs in a training session or match.

While the focus of this study is on the high-speed running activity as measured by HSR21pMIN and DIST21pMIN, to assist certain discussions we also utilize an additional metric Total DISTpMIN (Total distance covered by a player per minute of play) as follows:

(3) $$\mathrm{Total}\;\mathrm{DISTpMIN}=\frac{\mathrm{Total}\;\mathrm{distance}\;\mathrm{covered}\;\mathrm{on}\;a\;\mathrm{given}\;\mathrm{day}}{\mathrm{Duration}\;\mathrm{of}\;\mathrm{activity}\;\mathrm{during}\;\mathrm{the}\;\mathrm{day}\;\left(\mathrm{minutes}\right)}$$

### 2.4. Statistical Analysis

To understand the differences in physical activity demands during matches and training sessions, two kinds of statistical analysis were analysed: The Analysis of Variance (ANOVA) analysis and the probability distribution analysis. Where necessary, the data is tested to have originated from a Normal Distribution or a Weibull Distribution by utilising the Lilliefors Test [25].

The ANOVA analysis is utilised to compare the mean values of different populations. The objective of the ANOVA analysis is to determine if the differences in the mean values observed in a sample of data is due to sampling or not. The *p*-value signifies the probability to observe an extreme difference in mean because of the effect of sampling. In this study, ANOVA analysis was utilised to compare the mean value of HSR21pMIN variable between match sessions and training sessions. Furthermore, similar ANOVA analysis is performed on DIST21pMIN variable.

**Figure 1:**
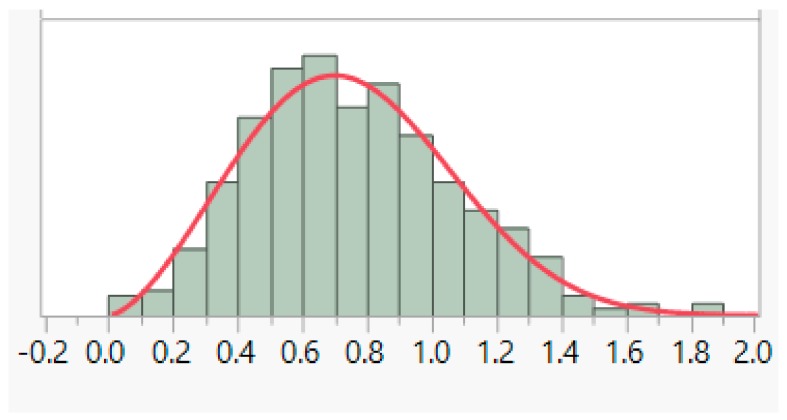
Weibull distribution fit to number of HSRs above 21 kmph per minute of play (HSR21pMIN) of all Full Backs (FB)/Central Forwards (CF)/Central Midfielders (CM) players, scale (*β*) = 0.848, and shape (*α*) = 2.566. *x*-axis: value of HSR21pMIN, *y*-axis: relative likelihood.

In addition to the ANOVA analysis to compare different physical activity demands, the probability distribution of per-session demand is analysed. The probability distribution analysis uses a statistical function to describe all the possible values and likelihoods that a random variable can take within a given range, which is bounded by minimum and maximum possible values. For this analysis, a suitable probability distribution function is fitted to a subset of physical activity data for the entire season. Three candidate probability distribution functions were utilised: Normal Distribution, Weibull Distribution, and the Log-normal Distribution and selected the best fit with the aid of a suitable goodness of fit measure. The Weibull Distribution was consistently a good fit with the data, and hence to simplify presentation, each data cluster with Weibull Distribution only is described. The Weibull Distribution of a random variable *x* is defined as in (4),

(4) f(x,α,β)={αβ(αβ)α−1e−(xβ)αx≥00x<0

where *α* is the shape parameter and *β* is known as the scale parameter. An example fit of the Weibull distribution to HSR21pMIN data is illustrated in Figure 1. The area under the curve of a probability density function is used to calculate the probability of an event occurring. Note that the probability density for any random variable could be more than 1, but the total area under the density curve equates to 1.

All statistical procedures were completed using MATLAB R2015b [26] and JMP 13.2.1 [27].

## 3. Results

### 3.1. Comparison of Average Match Demands Across Seasons and Positions

Descriptive statistics of physical activity demand measured by the number of high-speed runs (HSR21pMIN) and high-speed distance (DIST21pMIN) and total distance (Total DISTpMIN) are summarised in Table 2. To highlight the variations of activity demand associated with different playing positions, the statistics are presented for three different positions: Central Midfielders (CM), Centre Forwards (CF), and Full Backs (FB) across four seasons.

According to Table 2, there are several variations in the average number of high-speed runs and distance covered in high-speed runs across different seasons and positions. For example, the number of high-speed runs for FBs is different from CMs and CFs across all the seasons. Similarly, seasonal variations can be observed for a given position. For example, the average number of HSRs per minute for CFs increased from 0.79 in the 2014–2015 season to 1.39 in the 2017–2018 season. After a steady increase from 2014–2015 to 2016–2017, the demand for HSRs per minute in 2017–2018 for CMs and FBs remained fairly constant compared to the previous season for these positions. Different observations can be made for distance travelled during HSRs. For example, across all four seasons CFs tend to cover more distance at high speeds, and perform more HSRs than CMs and FBs. However, the Total DISTpMIN variable is higher for CMs as compared to CFs and FBs. Therefore, while CMs do the least number of high-speed runs compared to CFs and FBs, on average the CMs tend to cover more distance than CFs and FBs. Furthermore, in contrast to high-speed runs and high-speed distance, the average value for the total distance covered shows no variation across the seasons. Furthermore, the standard deviation of the Total DISTpMIN and the DIST21pMIN variable shows a downward trend across the seasons. This means that the distance covered by players has become more consistent across the seasons.

The match demands for the number of high-speed runs, high-speed distance, and total distance covered show variations across seasons and playing positions. However, in terms of the average values of HSRs and distance at high speeds, a consistent pattern has not been observed when all positions, seasons, and age groups are considered. In the next two sections, the patterns of training load variation across positions to cater for the complex match demands are analysed.

### 3.2. Comparison of Activity Level Demands during Competitive Matches and Training Sessions

**Figure 2:**
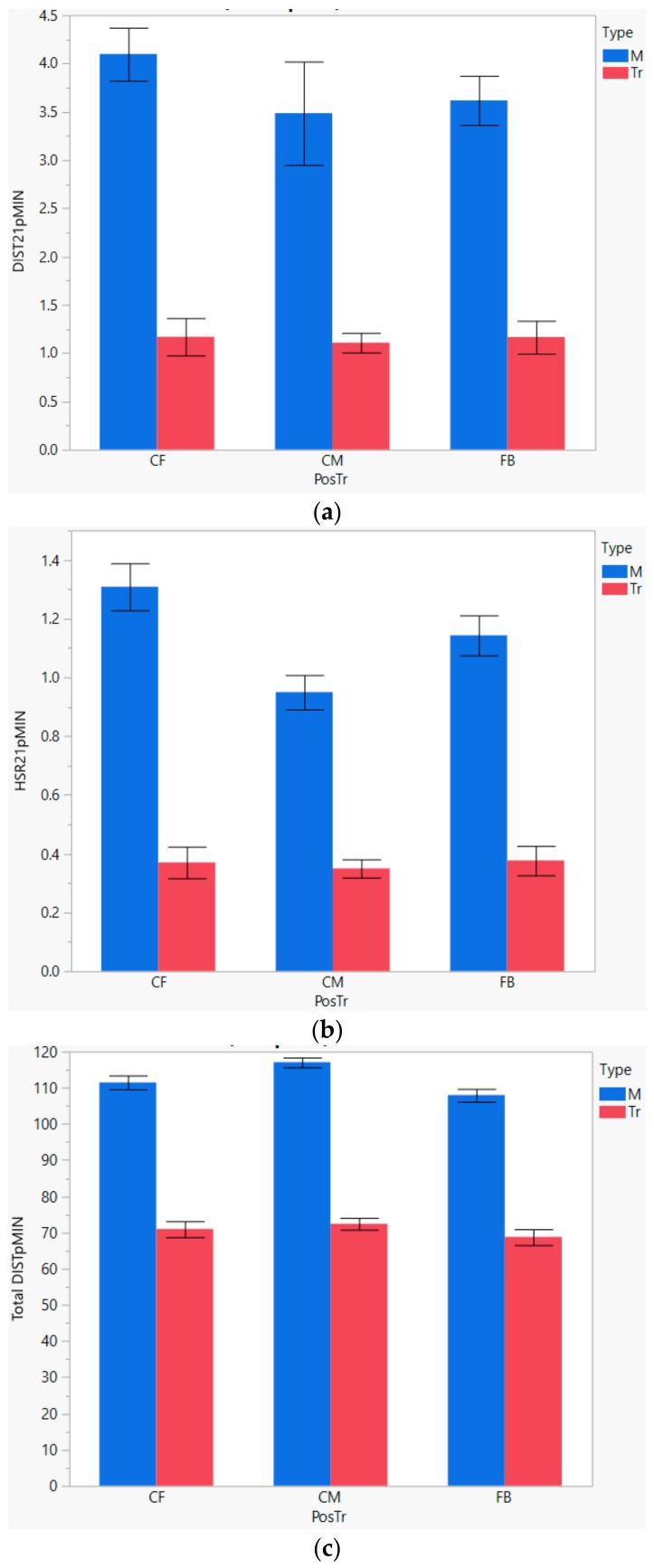
Comparison of mean physical activity demand for match sessions and training sessions for different positions of play for under 23 players (M: match, Tr: training). (**a**) DIST21pMIN; (**b**) HSR21pMIN; (**c**) Total DISTpMIN.

The comparison of mean values for different positions of play during match and training settings are illustrated in Figure 2. The error bars in the figure correspond to the 95% confidence interval of the mean. The mean values are compared utilising the one-way ANOVA analysis.

The HSR21pMIN variables show a significant difference between match and training session demands for all considered positions of play (CF: 1.31 in matches vs. 0.337 in training, CM: 0.95 vs. 0.334, and FB: 1.14 vs. 0.326 at *p*-value < 0.001). Similarly, the distances travelled during high-speed runs illustrate a significant difference between the match and training demands. The HSR21pMIN variables show a significant difference between match and training sessions (CF: 1.31 in matches vs. 0.337 in training, CM: 0.95 vs. 0.334, and FB: 1.14 vs. 0.326 at *p*-value < 0.001).

For training sessions in a given season, according to the ANOVA analysis performed, there is no statistical difference in the physical demands at different positions, as measured by HSR21pMIN and DIST21pMIN at a significance level of 0.05. The average demands for high-speed runs during training sessions, as measured by HSR21pMIN, for CF, CM, and FB (0.337, 0.334, 0.326) are deemed similar (*p* value: 0.8817). The average demands for distance covered during high-speed running, at positions CF, CM, and FB are deemed similar (1.15, 1.19, 1.09, *p* value: 0.5290). However, in contrast the average physical performance measured by Total DISTpMIN is significantly different for CF, CM, and FB positions during training sessions at a significant level of 0.05 (70.98, 72.46, 68.79, *p* value: 0.0333).

In matches, the physical activity level as measured by HSR21pMIN for CFs, CMs, and FBs are deemed significantly different from each other (1.31, 0.95, 1.14, *p* value < 0.001). Similarly, the Total DISTpMIN variable for CFs, CMs, and FBs are significantly different from each other (111.5, 117.1, 107.98, *p* value < 0.001). However, in contrast, in matches the activity levels measured by DIST21pMIN (distance covered during high-speed runs) at positions CF/CM/FB are deemed similar (4.08, 3.48, 3.59, *p* value 0.2329).

**Figure 3:**
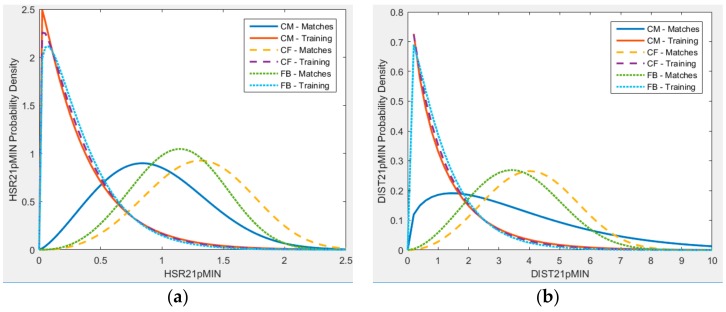
Probability density comparison of match day physical activity demand for different positions of play for under 23 players during matches and training sessions. (**a**) HSR21pMIN; (**b**) DIST21pMIN.

While there are differences in the mean values of physical activity parameters measured, a player would not experience the mean value of the activity in every match/training session during one season. Depending on the match situation, a player would have to perform at a higher activity level as compared to a different match. The distribution of the activity demand per-session of play (match or training) is shown in Figure 3 for the HSR21pMIN and DIST21pMIN variables. While there is no distributional difference in the training load variations for different positions of play, the match demand distribution exhibits a completely different behaviour, with CFs having to do comparatively more HSRs during a match. The activity demand as measured by HSR21pMIN for CMs for a match session is comparatively lower. For the DIST21pMIN variable, a similar pattern is observed, with similar training load distributions for different positions, while the match demand distribution is demonstrating variations. On average, the CMs have to cover a smaller distance at high-speed running with occasional demands for extremely high values as compared to CFs and FBs.

### 3.3. Patterns of Training and Match Demands Across Seasons

**Figure 4:**
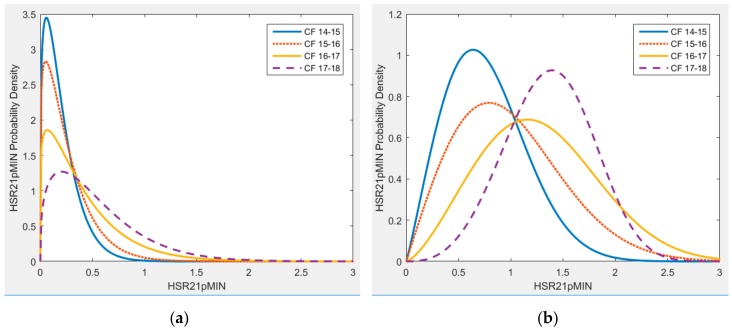
Probability density of high-speed running demands per minute of play, per-session for (**a**) training and (**b**) match sessions across four seasons for CFs.

**Figure 5:**
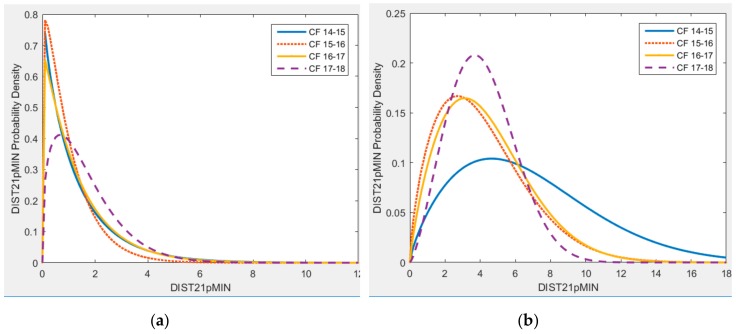
Probability density of distance covered during high-speed running per minute of play, per-session for (**a**) training and (**b**) match sessions across four seasons for CFs.

Figure 4 and Figure 5 illustrate the variation of probability distributions of activity demands per sessions across four seasons for the HSR21pMIN and DIST21pMIN variables. A similar pattern was also observed for the cases of CMs and FBs.

The activity demand during training sessions for HSR21pMIN clearly shows a distributional change across seasons, where the distribution mass has shifted towards the higher values of HSR21pMIN, as shown in Figure 4. This would mean that over the seasons, the probability of a player being exposed to a higher number of HSRs in the given sessions has increased. The distribution for demands during matches, while showing a different shape of distribution to training sessions, has also shifted towards the right.

The distributional differences for DIST21pMIN, as illustrated in Figure 5, show a different pattern. While there is a very little difference in the training demand distribution across the seasons, the distribution of match demands tends to concentrate more towards the lower values of DIST21pMIN. 

## 4. Discussion

The purpose of any training programme is to prepare players to perform in competition [28]. While there are studies that utilize player tracking data to compare between training and match sessions for sports such as rugby [28,29] and Australian rules football [30], there is very limited work conducted in relation to football (soccer) [31].

The aim of this study is to compare the physical activity demands in soccer between competitive matches and the training sessions across four seasons, with particular focus on positions of play. To the best of the authors’ knowledge, this is the first study to investigate the differences in activity demands during training and matches in relation to playing positions in football.

### 4.1. Positional Variation of High-Speed Running Activity during Training and Matches

There are significant positional differences (according to ANOVA analysis and probability distribution of per-session demand) within a season for the demand for HSRs during matches. The CFs face the highest demand for HSRs, compared to CMs and FBs. The CF is a central attacking position, whereas the role of FB is generally a more defensively orientated position in wide areas of the pitch.

On average the CFs cover a greater amount of high-speed running distance during matches, but ANOVA analysis on DIST21pMIN variable yields that there is no significant difference across the various positions of play. However, the probability distribution of activity demand per session shows that there are differences in the DIST21pMIN variable between positions of play.

According to the ANOVA analysis, the total distance covered during matches and training show significant positional differences. As illustrated in Figure 2, the CMs tend to cover the most distance as compared to CF and FB positions. The demand for total distance is comparatively higher for CFs as compared to FBs.

The role of a central midfielder is to contribute to both attacking and defensive phases of play. Although the total high-speed distance demands between FB and CM are similar, the frequency of high-speed runs in matches is higher in FB Position compared with the CM position (Figure 2).

The probability distribution of DIST21pMIN for CMs during matches shows that, although rare, occasionally the CMs have to cover relatively high levels of distance running at high speeds. The nature of the CM position may also mean these players cover significantly more total distance in comparison with other positions [3], and is in agreement with the results of this study as illustrated in Figure 2 and Table 2. The results of this study show significant differences in physical demands in matches across the varying positions played.

While there are significant positional differences during matches, ANOVA analysis yields that there are no positional differences in activity levels as measured by HSRs and distance covered during HSRs in training sessions. This could be due to other factors involved in team training sessions, as not all components of these sessions may be position-specific and may be based on general soccer skills. This observation is in agreement with the current literature which suggests that the external training load placed on soccer players tends to be similar due to the use of group training sessions [32]. Group training sessions can include technical and tactical components, which may take precedence over physical outcomes in order to prepare players for match play in soccer [32].

### 4.2. Comparison of High-Speed Running Activity between Training and Match Sessions

Furthermore, for all positions considered, the average value for HSRs and distance covered during HSRs are significantly different between the match and training sessions within a season. The demands during matches are always greater than those of the training sessions. Such finding is consistent with related research by Hartwig, Naughton, and Searl, which indicates that training physical activity levels are lower than match demands [29].

The major finding of this analysis as discussed in the preceding section is that while there are significant positional differences in physical activity demands during competitive matches, there is no such observable difference for activity demands during training. Further investigation into the physical outcomes from different types of training drills used, particularly those which are position-specific, could give a clearer insight into the relationship between training and matches, rather than looking at the outputs from training sessions as a total.

### 4.3. Seasonal Variation of Per-Session Activity Demands

For all three playing positions considered in this research (CF, CM, and FB), the average match demand for number of HSRs per-session has gradually increased over the four seasons. These findings for match demands are consistent with the literature, for example Lazarus et al. [33] reported that the number of HSRs in English Premier League football has increased over seven seasons. Based on the probability distribution fits, there is a gradual increase in the per-session demand for HSR during training sessions.

In contrast to HSRs, the distance covered in HSRs (DIST21pMIN variable) does not show significant seasonal variation for training sessions. However, for match sessions, the probability distribution fits suggest that there is a gradual decrease in the large values of DIST21pMIN over the seasons. Thus, when considering the seasonal variations of both HSR21pMIN and DIST21pMIN variables, there is a gradual tendency for a higher number of short HSRs. Variations in tactical efficiency, physical capabilities, playing style, formation of play, tournament demands, or any interaction between these factors could have contributed to this observation. Further analysis is necessary to understand the underlying reasons.

The seasonal training activity distribution for the HSR21pMIN variable corresponds to the variation in match activity. For example, a shift in the training load distribution towards the extreme values of HSRs has been observed across seasons, and for the same set of seasons, the match activity distribution has also shifted towards the higher values. Such correlation can be explained in more than one way: First, it could be because coaches conditioned players to perform more HSRs, which is reflected in match data. Secondly, it could be that different seasons posed different challenges, which prompted an increase in HSRs, or it could be a combination of these factors. Therefore, while causality cannot be inferred from these results to suggest that a distributional change in training load will affect a change in match activity, it opens a new avenue of investigation to find the relationship between training and match activity. For example, if a certain tournament that may demand a high number of HSRs is approaching, coaches may alter the training prescription in order to condition players for the forthcoming matches. Furthermore, patterns in position specific seasonal variations can lead to tailor made training programmes to cater to different positions of play.

### 4.4. Pointers for Further Study

While this study provides a macroscopic view of physical activity demands between matches and training, contextual factors such as different tournaments, playing formations, and game situations have not been considered. Furthermore, training sessions are not always physically-based, and involve different kinds of activities, such as technical skill training, high intensity activities, and game-based training sessions [28]. Analyses that consider such contextual variations would provide further insights into the differences between matches and training, as well as focusing on the varying types of training drills used within a session. Another important attribute that may account for the difference between matches and training is related to managing the fatigue of players. In periods of the season which are congested with competitive matches, the time between the games need to be carefully managed to stop over training of the players, which may lead to higher risk of injury. For example, during training sessions in-season, the training load may decrease when approaching a match day [31]. Therefore, microscopic analysis of training and match loads, for example on a weekly basis, would provide more contextual insights on how to adapt training programmes to position specific demands, while managing the fatigue of players [33].

Limitations of the current study warrant further research. Firstly, the seasonal comparison of activity demand distributions should ideally be normalised against the season specific demands. For example, a player may compete in different tournaments in different seasons. Different tournaments would require different levels of activity depending on the level of the opposition and the strategies employed [18]. Secondly, this analysis deals only with a certain age group (e.g., U23), and thus the findings cannot be generalised across different age groups. Future work may involve investigating tournament and age group induced variations on the difference between training and match sessions. Thirdly, looking into additional physical parameters derived from GPS tracking may highlight further positional differences in the physical demands imposed across training and matches.

## 5. Conclusions

GPS-based player tracking provides one of the most ubiquitous sources of data in football (soccer) and has the advantage of ease of collection over substantial amounts of time during competitions (i.e., match settings) and training sessions. The investigations presented in this paper illustrate how this data can be utilised to understand the physical activity demands placed on players during training sessions and in competitive matches.

The objective of this paper was to statistically compare the high-speed running activity demands between training and matches for different positions of play. The results of our study have indicated that, while there are significant position-specific differences in activity levels during matches, such differences are not observed for data pertaining to the training sessions. During matches, the attacking playing positions such as Centre Forwards (CF), consistently require players to perform higher number of high-speed runs (HSR: runs at speeds more than 21 kmph), compared to positions such as Central Midfielders (CM). However, from training session totals, there is no significant difference in the demand for HSRs at different positions. Similarly, the distance covered during HSRs, during training sessions show little or no positional variations, while there are certain positional differences during matches. Furthermore, the results of this study indicate that there are seasonal variations in the physical activity demands measured by HSRs or distance covered during HSRs. For the data set utilised, over four seasons, there is a gradual increase in the demand for HSRs, whereas the distance covered during HSRs tends to decrease. Although the results of this study provide interesting findings, further research considering other factors such as different tournaments, specific training drills, playing formations, in-season fatigue, and periodisation can provide more context and insight into the relationship between training load and matches. Therefore, analysing the tracking data with reference to drill-specific objectives as well as other contextual factors will be a focus of future work.

## Figures and Tables

**Table 1:** Number of players trained at different positions across seasons.

Season	Number of Players in Each Season				Total Number of Data Entries
Playing Position	2014–2015	2015–2016	2016–2017	2017–2018	
Centre Forward (CF)	13	14	12	9	4976
Centre Midfielder (CM)	18	22	20	19	10,896
Full Back (FB)	12	15	14	11	5041
Total	43	51	46	39	20,913

**Table 2:** Descriptive statistics for different playing positions for all four seasons during competitive matches, for the three metrics considered: HSR21pMIN, DIST21pMIN, and Total DISTpMIN.

	Season	2014–2015		2015–2016		2016–2017		2017–2018	
Playing Position	Metric	µ	σ	µ	σ	µ	σ	µ	σ
CF	HSR21pMIN	0.79	0.41	1.04	0.57	1.38	0.53	1.39	0.38
	DIST21pMIN	6.83	4.87	4.41	3.24	4.64	2.88	4.18	1.71
	Total DISTpMIN	111.6	21.34	110.1	18.14	112.4	13.5	110.2	11.17
CM	HSR21pMIN	0.65	0.70	0.81	0.52	1.02	0.47	1.15	0.72
	DIST21pMIN	5.66	7.56	3.48	2.93	3.76	3.25	3.70	2.30
	Total DISTpMIN	117.5	19.3	115.5	14.14	117.5	16.98	117.8	11.25
FB	HSR21pMIN	0.63	0.41	0.85	0.32	1.08	0.52	1.19	0.45
	DIST21pMIN	5.31	4.83	3.80	1.99	3.72	2.67	3.81	1.63
	Total DISTpMIN	108.7	13.2	108.4	15.12	107.6	11.2	108.5	9.47
